# Open Abdomen Treatment in Acute Pancreatitis

**DOI:** 10.3389/fsurg.2020.588228

**Published:** 2021-01-14

**Authors:** Jonas Henn, Philipp Lingohr, Vittorio Branchi, Alexander Semaan, Martin W. von Websky, Tim R. Glowka, Jörg C. Kalff, Steffen Manekeller, Hanno Matthaei

**Affiliations:** Department of General, Visceral, Thoracic and Vascular Surgery, University Hospital Bonn, Bonn, Germany

**Keywords:** acute pancreatitis, severe acute pancreatitis, abdominal compartment syndrome, risk analysis, open abdomen treatment

## Abstract

**Background:** Severe acute pancreatitis (SAP) is a heterogeneous and life-threatening disease. While recent guidelines recommend a stepwise approach starting with non-surgical techniques, emergency laparotomy remains inevitable in certain situations. Open abdomen treatment (OAT) may follow, potentially resulting in additional risks for severe morbidity. Causative factors and clinical impact of OAT in SAP are poorly understood and therefore issue of the present study.

**Materials and Methods:** A retrospective analysis of patients admitted to the Department of General, Visceral, Thoracic and Vascular Surgery at University of Bonn suffering from acute pancreatitis (ICD K.85) between 2005 and 2020 was performed. Medical records were screened for demographic, clinical and outcome parameters. Patients who received primary fascial closure (PFC) were compared to those patients requiring OAT. SAP-specific scores were calculated, and data statistically analyzed (*P* = 0.05).

**Results:** Among 430 patients included, 54 patients (13%) had to undergo emergency laparotomy for SAP. Patients were dominantly male (72%) with a median age of 51 years. Indications for surgery were infected necrosis (40%), suspected bowel perforation (7%), abdominal compartment syndrome (5%), and acute intra-abdominal hemorrhage (3%). While 22 patients (40%) had PFC within initial surgery, 33 patients (60%) required OAT including a median of 12 subsequent operations (SD: 6, range: 1–24). Compared to patients with PFC, patients in the OAT group had significantly fewer biliary SAP (*P* = 0.031), higher preoperative leukocyte counts (*P* = 0.017), higher rates of colon resections (*P* = 0.048), prolonged ICU stays (*P* = 0.0001), and higher morbidity according to Clavien–Dindo Classification (*P* = 0.002). Additionally, BISAP score correlated positively with the number of days spent at ICU and morbidity (*P* = 0.001 and *P* = 0.000002). Both groups had equal mortality rates.

**Discussion:** Our data suggest that preoperative factors in surgically treated SAP may indicate the need for OAT. The procedure itself appears safe with equal hospitalization days and mortality rates compared to patients with PFC. However, OAT may significantly increase morbidity through longer ICU stays and more bowel resections. Thus, minimally invasive options should be promoted for an uncomplicated and rapid recovery in this severe disease. Emergency laparotomy will remain ultima ratio in SAP while patient selection seems to be crucial for improved clinical outcomes.

## Introduction

Acute pancreatitis (AP) is a frequent cause of emergency admissions with rising incidence over the last years (1). Although various parameters have been identified contributing to onset and progression of disease, cholelithiasis and excessive alcohol consumption are the two predominant risk factors (2). According to the Atlanta Classification, AP clinically ranges from “mild” over “moderately severe” to “severe” (3). While most patients experience a rather uncomplicated course of disease, severe acute pancreatitis (SAP) is observed in 20–30 % of individuals with an alarming lethality of 15%. SAP is defined as a life-threatening clinical condition with persistent organ failure resulting from aggravated comorbidities (3, 4). In particular, the respiratory, cardiovascular, and renal systems are affected, why their functions need to be closely monitored in the course of disease (3). Moreover, extensive peripancreatic necrosis observed in up to 10% of AP patients harbors the risk for significant morbidity, especially in the presence of infection (5). In fact, controlling pancreatic necrosis and associated sepsis has been an essential task in SAP management and comprises the use of broad-spectrum antibiotics, radiological, and endoscopic interventions, as well as surgical measures (6).

Regarding the operative spectrum, early cholecystectomy is still highly recommended in patients with biliary pancreatitis. However, indication and optimal timing of other surgical interventions and especially of laparotomy, operative necrosectomy, and peritoneal lavage has been a matter of vivid debates (6). Generally, a “step-up” approach is nowadays favored for the treatment of SAP ranging from initial minimally invasive interventions such as CT or endoscopic drainage to open surgery reserved for complicated cases (7). In fact, various pathophysiological mechanisms of SAP result in essentially three main indications for emergency laparotomy: Firstly, SAP frequently induces systemic inflammatory response syndrome (SIRS), resulting in increased vascular permeability, the urge for excessive intravenous fluid substitution, and generalized edema while on intensive care unit (ICU). This in turn leads to high intraperitoneal pressure (IAP) culminating in an abdominal compartment syndrome (ACS), which is regularly treated with decompressive laparotomy (8, 9). Furthermore, acute intra-abdominal hemorrhage and otherwise not controllable infected necrosis are two other frequent causes for open surgery (10).

Whenever primary fascial closure (PFC) within initial surgery is impossible or unintended, patients need to undergo subsequent scheduled operations within “open abdomen treatment” (OAT). OAT relates to intentionally dispensing fascial approximation to allow continuous observation and treatment of intra-abdominal disease. Obvious advantages are paralleled by significant clinical risks such as abdominal wall hernia, entero-atmospheric fistulas, and increased mortality (11). Current guidelines support an individualized management stressing a meticulous selection of patients suitable for laparotomy and OAT (12) while an evidence-based approach reliably guiding surgical decision making in SAP is thus far not available. To stratify therapy decisions in SAP for an optimized outcome in this severe condition, we conducted single-center and retrospective analysis of patients from our tertiary referral center for pancreatic diseases who underwent operative therapy and, in part, OAT for SAP.

## Materials and Methods

### Patient Cohort

A retrospective analysis of patients admitted to the Department of General, Visceral, Thoracic and Vascular Surgery, University Hospital Bonn (Bonn, Germany), with the diagnosis of AP (ICD K.85) between 2005 and 2020 was performed. Patients with multiple admissions for AP were included as single cases respecting their first admission. Medical records were manually screened for patients that had undergone emergency laparotomy for SAP. Patients then were subdivided into two groups; firstly, patients who received PFC within their emergency laparotomy and secondly patients who received OAT. Demographic and clinical data (i.e., age at operation, gender, and comorbidities) were extracted, and the recent literature was scrutinized for additional relevant SAP and OAT-specific parameters to be included into our analysis. Etiologic factors for pancreatitis (i.e., cholelithiasis, alcohol, iatrogenic, and uncertain), pancreatitis-specific scores (i.e., BISAP, Atlanta), and relevant laboratory parameters [e.g., leukocyte count, (LC), c-reactive protein (CRP), procalcitonin (PCT), pancreas-specific lipase, and amylase] were documented (3, 13). Indications for emergency laparotomy (i.e., therapy-refractory infected necrosis, suspected bowel perforation, ACS, and acute hemorrhage) and reasons for OAT (i.e., peritonitis, fascial retraction, and other), as well as the numbers of subsequent reoperations, days of OAT, and OAT technique (vicryl mesh, visceral protection layer, and negative pressure) were charted. To determine the specific outcome of OAT, we assessed morbidity by Clavien–Dindo classification and evaluated the need for colon resection, the duration at ICU, and general hospitalization (14). Furthermore, in-house hospital mortality was calculated.

### Statistics

Descriptive and inferential statistics were used in data analysis using SPSS Statistics (IBM, Armonk, New York, USA). Intergroup differences were calculated using CHI-squared- and Student's *t*-test. Pre-, intra-, and postoperative parameters were analyzed for possible correlations using the Pearson correlation coefficient. Findings were compared with recent treatment guidelines and literature. *P*-values were 2-sided, and statistical significance was set at 0.05.

## Results

### Patients, Comorbidities, and SAP

Of 430 patients with AP treated between 01.01.2005 and 01.01.2020, 54 patients (13%) needed emergency laparotomy because of SAP. The PFC group included 22 (41%) patients and the OAT group 32 (59%) patients. Table 1 summarizes both groups, their composition, and their relevant comorbidities. Neither sex nor age showed significant intergroup differences (*P* > 0.05). With the exception of significantly more pulmonary diseases in the PFC group (*P* = 0.02), comorbidities did not differ significantly. While no patient of the PFC group had undergone previous laparotomy, four patients (13%) of the OAT group had a history of abdominal operations.

**Table 1:** Baseline characteristics of all patients, divided into PFC and OAT group.

	**PFC group**		**OAT group**		***P***	**OR/mean difference**	**CI95**	
All (*n*, %)	22	41	32	59				
Sex m (*n*, %)	15	68	23	72	0.716	1.193	0.365	3.892
Sex w (*n*, %)	7	32	9	28	0.716	0.839	0.257	2.736
Age (mean days, range)	54	26–74	49	25–80	0.221	4.591	−2.863	12.045
Any comorbidity (*n*, %)	17	77	23	72	0.657	0.752	0.213	2.650
Hypertension (*n*, %)	10	45	12	38	0.559	0.720	0.239	2.169
Cardiac disease (*n*, %)	7	32	7	22	0.413	0.600	0.176	2.048
Diabetes (*n*, %)	6	27	4	13	0.170	0.381	0.093	1.555
Hepatic disease (*n*, %)	4	18	6	19	0.958	1.038	0.256	4.214
Malignant disease (*n*, %)	1	5	3	9	0.506	2.172	0.211	22.368
Pulmonary disease (*n*, %)	5	23	1	3	**0.024**	**0.110**	**0.012**	**1.017**
Renal disease (*n*, %)	0	0	0	0	–	–	–	–
Other (*n*, %)	13	59	13	41	0.182	0.474	0.157	1.429
Previous laparotomy (*n*, %)	0	0	4	13	0.085	1.429	0.238	8.571
Cause								
Uncertain (*n*, %)	2	9	10	31	0.054	4.545	0.887	23.304
Iatrogenic (*n*, %)	3	14	3	9	0.624	0.655	0.119	3.592
Cholelithiasis (*n*, %)	11	50	7	22	**0.031**	**0.280**	**0.086**	**0.915**
Alcohol (*n*, %)	6	27	12	38	0.433	1.600	0.492	5.207
LC (mean G/l, range)	12	2–28	17	5–46	**0.015**	**5.586**	**1.15**	**10.02**
CRP (mean mg/l, range)	129	25–215	195	74–347	**0.048**	**66.045**	**0.61**	**131.48**
PCT (mean μg/l, range)	3	0–8	36	0–345	0.304	33.610	−35.404	102.624
Lipase (mean U/l, range)	233	41–809	676	3–5191	0.169	442.995	−202.901	1,088.892
Amylase (mean U/l, range)	68	22–170	113	7–770	0.254	44.170	−33.415	121.756
BISAP (mean, range)	2	0–4	2	0–4	0.179	0.491	−0.235	1.218
Atlanta (mean, range)	2	1–3	3	2–3	0.200	0.199	−0.109	0.507
Indication								
Infected necrosis (*n*, %)	18	82	21	66	0.567	0.424	0.115	1.566
ACS (*n*, %)	0	0	5	16	0.056	–	–	–
Bowel perforation (*n*, %)	2	9	5	16	0.509	1.852	0.325	10.538
Acute hemorrhage (*n*, %)	2	9	1	3	0.332	0.323	0.027	3.796
Colon resection (*n*, %)	3	14	8	25	**0.048**	**2.111**	**0.492**	**9.063**
Enteric stoma (*n*, %)	1	5	8	25	**0.032**	**7.000**	**0.807**	**60.684**
Hospital stay (mean days, range)	65	8–184	84	24–217	0.116	19.043	−4.949	43.034
ICU stay (mean days, range)	14	0–100	45	3–120	**0.001**	**31.097**	**15.85**	**46.34**
Clavien–Dindo (mean, range)	3	1–5	4	3.5–5	**0.002**	**1.078**	**0.45**	**1.71**
Mortality (*n*, %)	3	14	1	3	0.138	0.204	0.020	2.108

*For dichotomous variables the odds ratio (OR) and for continuous variables, the mean difference is stated. CI 95=95% confidence interval. Characteristics with significant differences between the two groups are printed bold*.

While cholelithiasis (*N* = 18, 33%) and alcohol abuse (*N* = 18, 33%) accounted for the most frequent etiologic factors, iatrogenic pancreatitis occurred in six patients (11%). In the remainder, the exact cause remained elusive (*N* = 12, 22%). In the OAT group, biliary SAP was significant less frequent (*P* = 0.03) and there was a tendency for more unclarified etiologies (*P* = 0.054). Emergency laparotomy was in most patients performed for therapy-refractory-infected necrosis (*N* = 39, 72%). Suspected bowel perforation with free intra-abdominal air on CT scans was indication for surgery in seven patients (13%). Decompressive laparotomy for ACS was required in five patients (9%), and operative exploration for acute severe hemorrhage in three individuals (6%). While ACS showed a trend to lead to OAT at a higher rate (*P* = 0.055), no such coherences were observed for other surgical indications. Compared to the PFC group, in OAT patients preoperative LC and CRP levels were significantly increased (*P* = 0.015 and *P* = 0.048), whereas no differences were observed for other serum parameters such as PCT, lipase, and amylase. Median BISAP scores for PFC and OAT groups were 1.5 (range 0–4) and 2 (range 0–4), and median Atlanta Classification was 2 (range 1–3) and 3 (range 2-3), respectively. No significant differences were measured regarding both scores.

### Post-laparotomy Treatment and Outcome

Operation protocols stated two distinct reasons leading to OAT: Most frequently, severe peritonitis with abdominal sepsis present in 12 patients (38%) demanded second-look operations and prevented the surgeon from closing the abdominal cavity. The second cause was fascia retraction and dehiscence precluding primary fascial closure which was found in eight individuals (25%); including five cases in which ACS triggered initial laparotomy. A median of 12 subsequent reoperations were necessary in the OAT cohort, resulting in a median of 27 days until the final surgical intervention. Intestinal damage was a recurring complication (*N* = 11, 20%) and significantly higher in the OAT cohort reflected by more colon resections (*P* = 0.048) and a higher rate of enteric stomata (*P* = 0.032). Abdominal wall was temporarily closed with vicryl mesh interposition in 17 OAT patients (53%) of which 4 (13%) were supported with a protective visceral layer and 2 (6%) with vacuum wound therapy. Most patients (*N* = 22, 569%) were dismissed with planned ventral hernia (e.g., dry secondary wound closure, suture of skin above secondary healing abdominal wall), whereas delayed primary closure was performed in 10 patients (31%).

**Figure 1:**
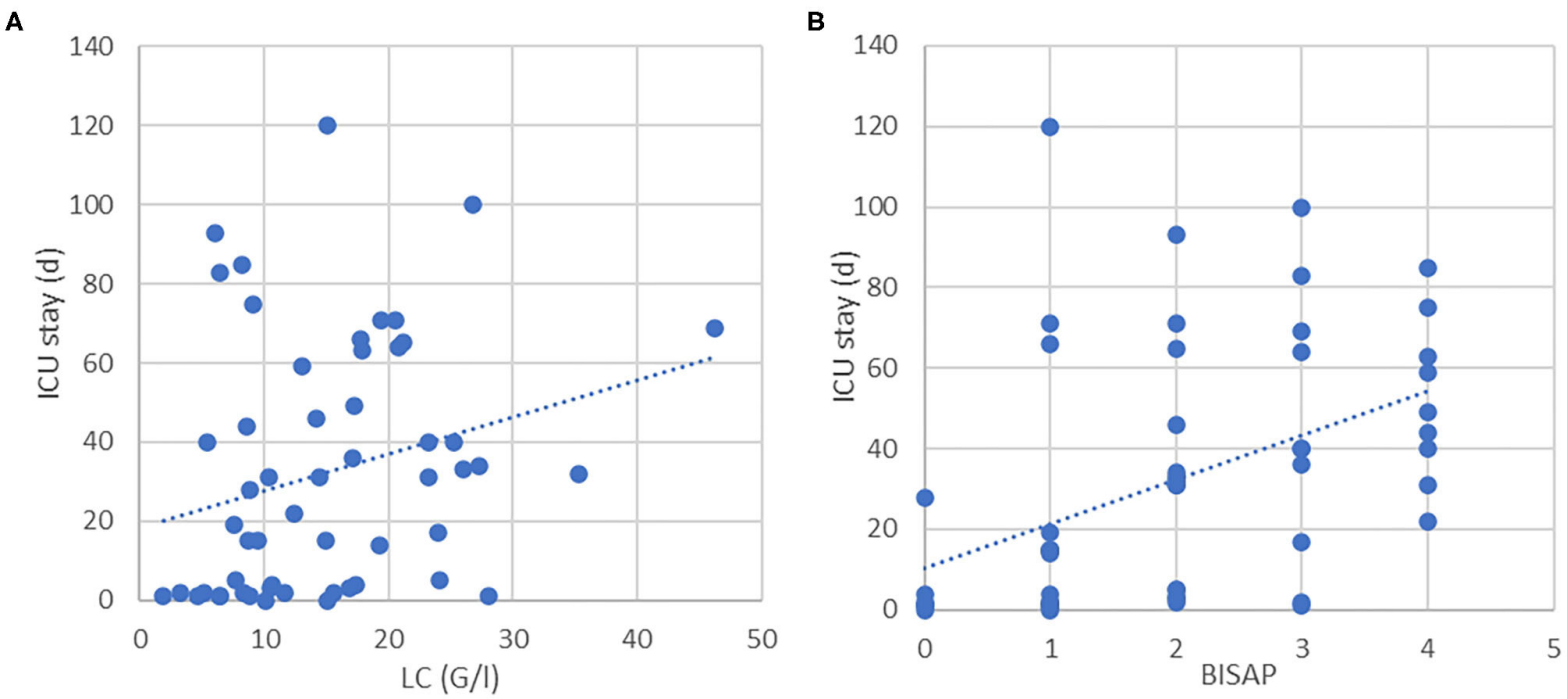
**(A)** Correlation between LC and ICU stay (*P* = 0.062). **(B)** Correlation between BISAP and ICU (*P* < 0.001).

Median ICU stay was 14 days and 45 days for the PFC and the OAT group, respectively, with significantly longer ICU stays in the OAT cohort (*P* = 0.0002). As shown in Figure 1, LC and BISAP correlated positively with the lengths of ICU stays; however, only correlation between BISAP and ICU stay was statistically significant (*P* = 0.06 and *P* = 0.001). SAP patients were hospitalized for a median of 71 days (range 8–217). Despite longer ICU treatment, OAT patients did not spend longer time in hospital compared to PFC patients (*P* = 0.12). An overall longer hospital stay was not significantly related to any preoperative parameter included. None of the remaining preoperative parameters correlated relevantly with length of ICU stay or overall hospitalization.

**Figure 2:**
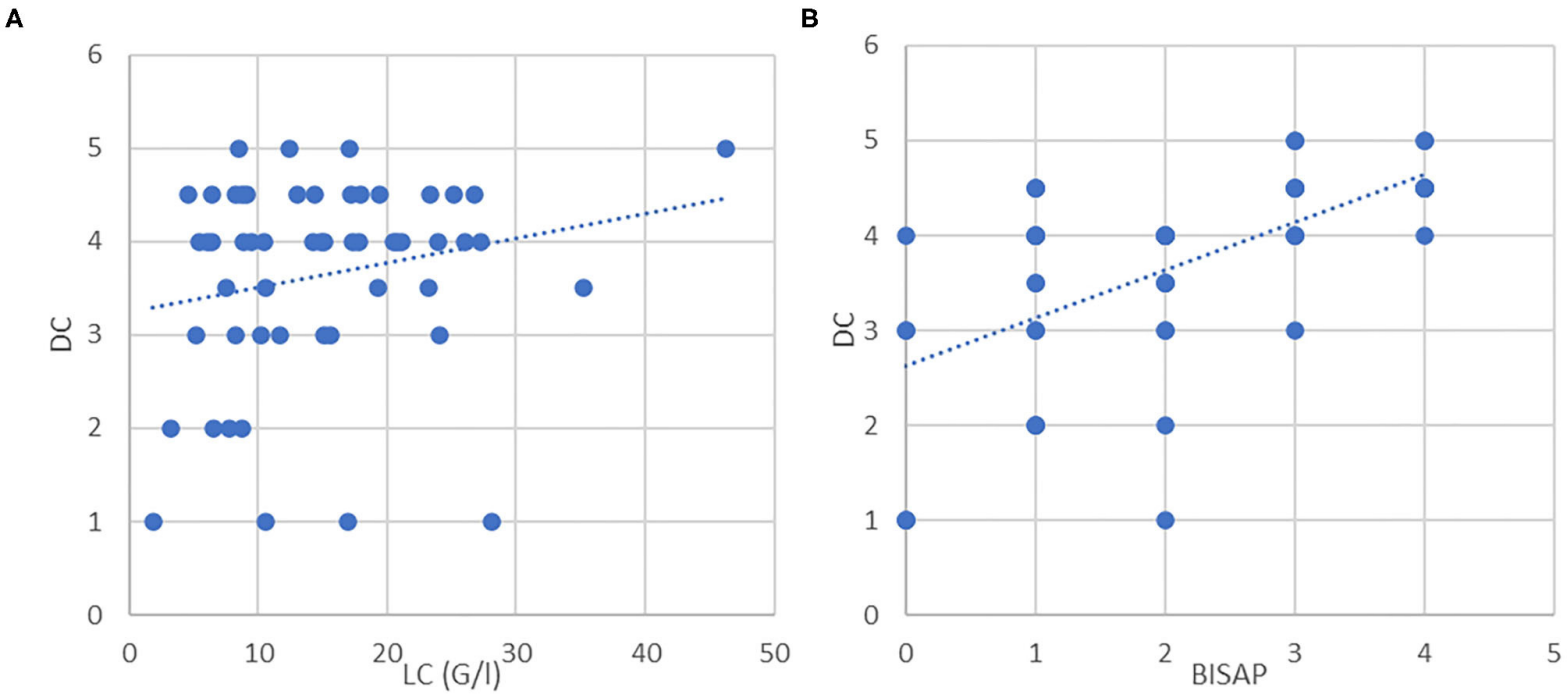
**(A)** Correlation between LC and DC (*P* = 0.122). **(B)** Correlation between BISAP and DC (*P* < 0.001).

Overall, the mean grade of complication (or morbidity) according to Clavien–Dindo classification was 4 (range 1–5), while the OAT group had increased morbidity with significantly higher Clavien–Dindo scores (*P* = 0.0015). LC and BISAP showed positive correlation with Clavien–Dindo classification Figure 2 with significance for BISAP score. All other parameters showed no relevant impact on morbidity.

Overall mortality was 7% (*N* = 4) and showed no intergroup difference. The small number of patients precluded valid statistical analyses for risk factor analysis. Deceased patients were all male and had a median age at operation of 49 years (range 29–74). Half had no prior comorbidities (50%) with varying causes for SAP [alcohol (*N* = 2), bile stone (*N* = 1), and iatrogenic (*N* = 1)]. Cause of death was sepsis triggered multi-organ failure in all four patients.

## Discussion

The incidence of AP is currently on the rise in western countries while particularly elderly patients are affected with most individuals being in their 70s (15). Not surprisingly, a higher disease-related mortality could be evidenced in these compared to younger patients (16). In contrast, it is the middle-aged adults who were reported to have the highest risk for aggravated disease, which is in concordance with the mean age of 52 years in our analysis (17). Furthermore, evidence is emerging that preexisting comorbidities rather than age *per se* seem to trigger adverse outcomes. The demographic shift will increase the number of endangered patients and therefore we should expect AP to gain further clinical relevance with a potentially growing financial burden to our health care system both justifying scientific dedication to that topic (1).

Adequate treatment of AP is laboriously studied, and distinct focus lies on the prevailing question when to intervene in SAP since any unnecessary therapy might cause further significant morbidity in these severely ill patients. Especially, periprocedural trauma caused by abdominal surgery results in increased risk for severe morbidity and mortality and therefore needs to be avoided (18). Thus, minimally invasive approaches such as endoscopic and interventional radiological techniques have been developed over the past two decades and resulted in the least trauma for maximum impact (19). When comparing different interventions, three randomized controlled trials all favored endoscopic over percutaneous drainage. On the other hand, inconsistent results were reported when comparing endoscopic with surgical treatment of infected necrosis (7, 20, 21).

### Surgical Treatment

Emerging evidence in this clinical field has still not provided precise algorithms unequivocally guiding surgical therapy in SAP. Thus, indication for surgery is heavily debated upon and depends on the experience and preference of the interdisciplinary team. In concordance to Jacob et al. therapy-refractory-infected necrosis was the major cause for laparotomy in our cohort followed by suspected bowel perforation, ACS, and acute intra-abdominal hemorrhage (22). Supporting recent recommendations, our findings stress the need for sparing certain patients with infected necrosis from surgery to reduce periprocedural morbidity. However, recent guidelines recommend decompressive laparotomy as treatment of choice in cases of ACS. Here a rapid laparotomy is frequently unavoidable and is sometimes performed at ICU, for example, if the patient transport appears risky (9). Since our meticulous literature study failed to reveal any relevant data on bowel perforation in SAP, we can only share our single-center experience. As in other conditions, surgery is the standard treatment in case of free abdominal air why percutaneous drainage merely can be performed as a bridging strategy before definitive surgical treatment.

The clinical concept of OAT emerged over the past three decades, while surgeons left the dogma of PFC in favor of benefits associated with the open abdomen. In general, two main scenarios lead to an inability to perform PFC: on the one hand, generalized edema leads to fascial dehiscence in which approximation is impossible and the surgeon is left with virtually no choice. This scenario is typically applied in ACS. On the other hand, definitive closure of the abdominal cavity is postponed due to the need for revision operations. Here, consideration is heavily based on the experience of surgical team and therefore remains poorly objectifiable. While fortunately continuous improvements broadened our spectrum of surgical therapy in these critically ill patients, our findings suggest that laparotomy and OAT independently contribute to morbidity (23). Unsuitably, even repetitive fascial approximation may lead to secondary wound healing. To allow a timely fascial closure and to prevent formation of a large ventral defect, various techniques have been reported. Mesh-mediated fascial traction represents one of the most used techniques and is also standard at our center (24). Additionally, a combination of mesh-mediated fascial traction and negative wound pressure (e.g., vacuum assisted systems) is generally accepted as best practice, leading to highest rates of fascial closure (25). Hereby, the unacceptable high rates of ventral hernia can be decreased and therefore given techniques are highly recommended. For various reasons and especially in the early era, only half of our OAT patients were treated by this contemporary standard. Finally, innovative approaches such as “fasciotens” or “ABTHERA” are currently being evaluated to allow complete fascial closure before demission (26, 27).

Despite all dedication to improved results, OAT significantly increases risk for morbidity when compared to PFC and secondary morbidity following ICU treatment and possible extensive reconstructive operations must be taken into consideration additionally if the attempt at delayed primary fascial closure fails. Reported mortality in surgically treated SAP patients ranges up to a horrifying 65% (28). Overall, we detected a much lower rate which may indicate a safe procedure when patients are carefully selected. In literature, OAT patients independently from genesis were received less excessive surgical treatment than our OAT patients. This may be seen as another expression of the unfavorable combination of SAP and surgical interventions (23, 29, 30).

### Risk Factor Analysis

Until now, there are no established risk factors to determine in an early phase which patients will benefit from surgery or likely require OAT. Starting with demographic factors, reported increased mortality above the age of 70 cannot be supported by our data. In contrast to large population-based studies, two thirds of our SAP cohort are represented by men and therefore male sex seems to represent a decisive factor for severity of AP (31). Moreover, etiology-specific outcomes have been observed in AP and, in line with recent literature, bile stones and alcohol were the most common causes for SAP in our cohort. Previous data linking alcohol-induced AP to higher morbidity and mortality could not be supported by our study (32). Individuals in the OAT group had fewer cholelithiasis-induced SAP and suffered more often from idiopathic disease, although the latter did not reach statistical significance. This finding is in line with Zhu et al. who evidenced that AP caused by bile stones shows milder disease while severe and complicating diseases were mainly observed in idiopathic cases (33). The more favorable course of biliary pancreatitis can be related to the opportunity for eliminating the causative factors through ERCP/cholecystectomy allowing a rapid and full recovery. Since biliary pancreatitis is more common in women, data suggest female sex as a protective factor for OAT (34).

Because BISAP score was designed to predict mortality in SAP and showed its efficiency in a large population-based study (13), we evaluated the score‘s use in prediction of OAT. Though BISAP correlated significantly with ICU stay and Clavien–Dindo classification in both groups and therefore seems to be an appropriate marker for morbidity, no significant difference in BISAP was observed between the OAT and PFC groups. Although LC represents an unspecific value, it differentiated the most between both groups and may therefore indicate or even predict OAT. Moreover, Stirling et al. conducted a retrospective study for severity stratification in SAP patients and concluded that large changes in, and excessive, CRP levels predict severe disease (35). Accordingly, our OAT group expressed significantly higher CRP levels, suggesting that CRP may indicate a more critical course suggesting the need for OAT. Our data could not confirm the usefulness of PCT in severity prediction, which is in line with inconsistent results reported in literature (36).

For further validation of presented and identification of additional risk parameters of OAT in SAP, prospective randomized multicenter trials are needed. With ever growing medical data, new bioinformatic techniques of data analysis (e.g., artificial intelligence) seems most appropriate for this urgent task.

### Limitations

We acknowledge several limitations in our study, mainly with respect to study design and patient cohort. The retrospective design and the relatively small number of patients, even if treated at our tertiary referral center for pancreatic diseases, reduce the level of evidence of our findings. Additionally, missing clinical data reasoned by a study period of 15 years further limits the statistical power. Furthermore, data was retrieved from a single center, with the potential risk for selection bias. Since OAT is a therapeutic concept underlying constant improvements, no uniform standard has been applied in this historic cohort hampering direct comparison of treatment. To provide sufficient evidence for this crucial field, future studies need to leverage the power of created registries (37).

### Conclusion

Our data suggest that preoperative factors in surgically treated SAP may indicate the need for OAT and predict the postoperative outcome. Using these parameters, OAT patients may in the future ideally be triaged, and their management scheduled in an early phase. OAT itself appears to be a safe option with equal mortality and hospital stay compared to PFC. However, OAT may significantly increase morbidity with longer ICU stays and higher chance of bowel resection. A minimally invasive step-up approach has been shown superior to open surgery for the treatment of SAP. In combination with presented data, we suggest avoiding open surgery and particularly OAT in the treatment of SAP, whenever possible. Besides clear indication, e.g., for ACS and bowel perforation, emergency laparotomy remains the ultima ratio in SAP-related infections and evidence-based indications should be aimed in future for best patient outcomes. OAT cannot entirely be eliminated in the interdisciplinary management of SAP why respective knowledge and technical skills are mandatory for the abdominal surgeon.

## Data Availability Statement

The raw data supporting the conclusions of this article will be made available by the authors, without undue reservation.

## Ethics Statement

Ethical review and approval was not required for the study on human participants in accordance with the local legislation and institutional requirements. Written informed consent for participation was not required for this study in accordance with the national legislation and the institutional requirements.

## Author Contributions

JH, MW, and HM contributed to conception and designed the study. JH and HM drafted the manuscript. All the authors contributed to manuscript revision and approved the manuscript.

## Conflict of Interest

The authors declare that the research was conducted in the absence of any commercial or financial relationships that could be construed as a potential conflict of interest.
